# Effect of Temperatures on Drying Kinetics, Extraction Yield, Phenolics, Flavonoids, and Antioxidant Activity of *Phaleria macrocarpa* (Scheff.) Boerl. (Mahkota Dewa) Fruits

**DOI:** 10.3390/foods12152859

**Published:** 2023-07-27

**Authors:** Fatin Nurain Stephenus, Mohammad Amil Zulhilmi Benjamin, Adilah Anuar, Mohd Azrie Awang

**Affiliations:** 1 Faculty of Food Science and Nutrition, Universiti Malaysia Sabah, Jalan UMS, Kota Kinabalu 88400, Sabah, Malaysia; 2 Borneo Research on Algesia, Inflammation and Neurodegeneration (BRAIN) Group, Faculty of Medicine and Health Sciences, Universiti Malaysia Sabah, Jalan UMS, Kota Kinabalu 88400, Sabah, Malaysia; 3 Faculty of Chemical Engineering Technology, Universiti Malaysia Perlis, Kampus UniCITI Alam, Sungai Chuchuh, Padang Besar 02100, Perlis, Malaysia; 4 Innovative Food Processing and Ingredients Research Group, Faculty of Food Science and Nutrition, Universiti Malaysia Sabah, Jalan UMS, Kota Kinabalu 88400, Sabah, Malaysia

**Keywords:** *Phaleria macrocarpa*, drying kinetics, extraction yield, phenolics, flavonoids, antioxidant activity, effective moisture diffusivity, activation energy

## Abstract

*Phaleria macrocarpa* (Scheff.) Boerl. or ‘Mahkota Dewa’ is a popular plant found in Malaysia as it is a valuable source of phytochemicals and therapeutic properties. Drying is an essential step in the storage of *P. macrocarpa* fruits at an industrial level to ensure their availability for a prolonged shelf life as well as preserving their bioactive compounds. Hence, this study evaluates the effect of different temperatures on the drying kinetics, extraction yield, phenolics, flavonoids, and antioxidant activity of *P. macrocarpa* fruits. The oven-drying process was carried out in this study at temperatures of 40 °C, 50 °C, 60 °C, 70 °C, and 80 °C. Six thin-layer drying models (i.e., Lewis, Page, Henderson and Pabis, two-term exponential, Logarithmic, and Midilli and Kucuk models) were evaluated to study the behaviour of oven-dried *P. macrocarpa* fruits based on the coefficient of determination ($R^{2}$), root mean square error (RMSE), and chi-square ($\chi^{2}$). The quality of the oven-dried *P. macrocarpa* fruits was determined based on their extraction yield, total phenolic content (TPC), total flavonoid content (TFC), and antioxidant activity (2,2-diphenyl-1-picrylhydrazyl) using ultrasound-assisted extraction. The results showed that the time for moisture removal correspondingly increased in the oven-dried *P. macrocarpa* fruits. Apparently, the Midilli and Kucuk model is the most appropriate model to describe the drying process. The range of effective moisture diffusivity was 1.22 × $10^{-8}$ to 4.86 × $10^{-8}$ $m^{2}/s$, and the activation energy was 32.33 kJ/mol. The oven-dried *P. macrocarpa* fruits resulted in the highest extraction yield (33.99 ± 0.05%), TPC (55.39 ± 0.03 mg GAE/g), TFC (15.47 ± 0.00 mg RE/g), and DPPH inhibition activity (84.49 ± 0.02%) at 60 °C based on the significant difference (*p* < 0.05). A strong correlation was seen between the antioxidant activity, TPC, and TFC in the oven-dried *P. macrocarpa* fruits. The current study suggests that the oven-drying method improved the TPC, TFC, and antioxidant activity of the *P. macrocarpa* fruits, which can be used to produce functional ingredients in foods and nutraceuticals.

## 1. Introduction

**Figure 1:**
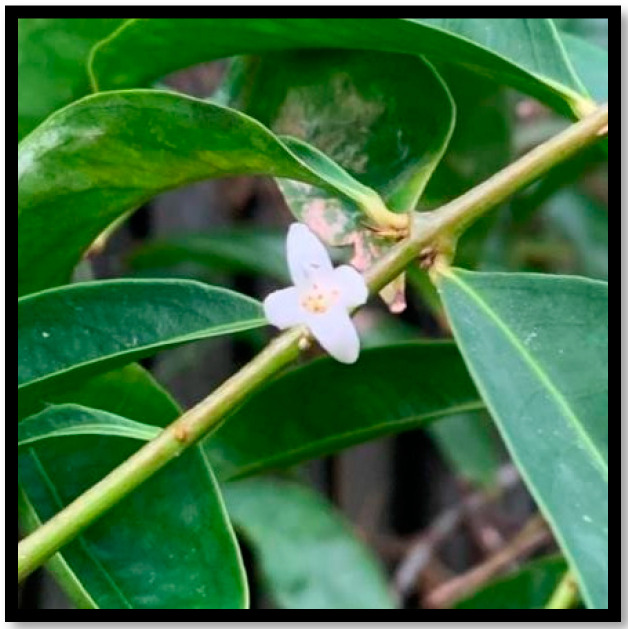
*P. macrocarpa* flower.

**Figure 2:**
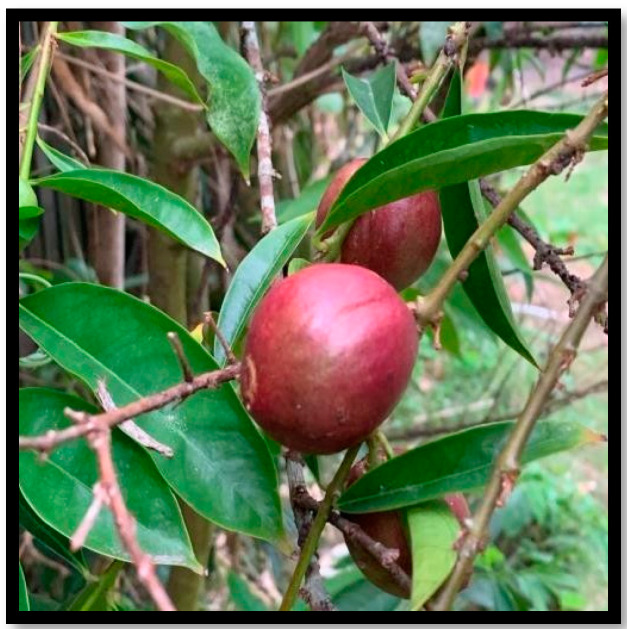
*P. macrocarpa* fruits.

Plant-derived pharmaceuticals are becoming more popular, as reflected by the growth in pharmaceutical industries that produce these herbal medications [1]. Herbal medications are increasingly used for various reasons, including their ease of availability, lower prices than modern treatments, and their perceived therapeutic value [1]. *Phaleria macrocarpa* (Scheff.) Boerl. is one of the medicinal plants that has become overwhelmed in recent years [2]. Known as ‘Mahkota Dewa’, this Thymelaeaceae family plant can be found in Indonesia and Malaysia [2]. Initially, it was believed that the fruits are dangerous and highly toxic [2]. *P. macrocarpa* fruits are widely utilised in traditional medicine as numerous advantages were continuously discovered [2]. Typically, *P. macrocarpa* fruits and leaves were traditionally used to treat cancer, diabetes mellitus, and hypertension [3]. It is also mixed with other medicinal plants as a potential treatment [3]. Dried *P. macrocarpa* fruits were also reported to have various beneficial phytochemicals such as phenolics, flavonoids, saponins, alkaloids, phytosterols, and tannins [4]. According to previous research, the fruits also contain antioxidant, anti-inflammatory, antihypertensive, antidiabetic, antibacterial, antifungal, vasorelaxant, and cytotoxicity properties [2]. Hence, it is highly possible that *P. macrocarpa* fruits could promote disease prevention and serve several health benefits. Figure 1 and Figure 2 shows the flower and fruits of *P. macrocarpa*, respectively.

Drying is one of the most widespread and time-consuming methods used to maintain the freshness, flavour, and nutritional value of fruits and vegetables for longer periods of time. It allows for the preservation of compounds, prolonged shelf life, and enhancement in bioavailability in fruits and vegetables, significantly minimising microbial spoilage and deterioration reactions during management and storage [5]. Oven-drying is a standard method used for drying various plant materials [5]. It is one of the simplest and quickest thermal processing techniques that can preserve phytochemical content [6] and is extensively used in the food industry [5]. To find the best conditions for drying *P. macrocarpa* fruits is essential, and some parameters should be considered. The presence of natural antioxidants in certain fruits, which are typically believed to have an antioxidant capacity, prompts researchers to determine the optimal oven-drying temperature for protecting the flavonoids and phenolics of the fruits. Moreover, the complex process of drying kinetics requires the application of mathematical modelling to describe the drying behaviour of the dried items, as well as to estimate the drying duration of some products [7]. The model explains the process of water removal from porous media by evaporation through a thin material layer until the water content equilibrium is achieved [8]. Temperature, product thickness, drying duration, surface area, and relative air humidity are among the factors that may influence the drying kinetics [9]. Consequently, the use of mathematical models to simulate the kinetics and explain the mechanism of water transference in *P. macrocarpa* fruits is an effective method for controlling the process.

Considering all these facts, this study aims to evaluate the effect of the oven-drying conditions (temperatures) on the phenolics, flavonoids, and antioxidant activity in *P. macrocarpa* fruits in order to determine the optimal drying conditions that will permit a high-quality standard of the fruit for use as a raw material in the production of functional ingredients for foods and nutraceuticals. In addition, the drying kinetics of *P. macrocarpa* fruits were mathematically modelled to examine their behaviour and determine the most effective model. To the best of our knowledge, this study is the first time that the modelling of the drying process of *P. macrocarpa* fruits has been carried out at this range of temperatures (40–80 °C) using six different thin-layer drying models.

## 2. Materials and Methods

### 2.1. Chemicals, Reagents, and Equipment

The current study utilised methanol and ethanol that were purchased from HmbG (Hamburg, Germany). Folin–Ciocalteu (F–C) reagent, sodium carbonate (Na_2_CO_3_), gallic acid, aluminium chloride (AlCl_3_), rutin, ascorbic acid, and 2,2-diphenyl-1-picrylhydrazyl (DPPH) reagent were supplied by Merck (Darmstadt, Germany).

A convection drying oven (ED 23, Binder, Neckarsulm, Germany) and analytical balance (GR-200, A&D, Tokyo, Japan) were used during the drying process of *P. macrocarpa* fruits. Filter papers (Whatman, Maidstone, United Kingdom), electric blender (EBM-9182, Elba, Borso del Grappa, Italy), ultrasonic bath [CPX8800H, Branson, CT, USA (United States of America)], rotary evaporator (Laborota 4000, Heidolph, Schwabach, Germany), and ultraviolet–visible (UV–Vis) spectrophotometer (Lambda 25, Perkin Elmer, MA, USA) were utilised for the extraction and other analyses.

### 2.2. Collection of Raw Materials

An amount of 4 kg of fresh and ripened *P. macrocarpa* fruits were obtained in the early morning from vendors at Kota Kinabalu, Sabah, Malaysia (Coordinate: 6°03′32.1″ N 116°09′24.9″ E). The fruits were stored in the Post Harvest Technology Laboratory at the Faculty of Food Science and Nutrition, Universiti Malaysia Sabah. Then, *P. macrocarpa* fruits were washed using tap water to remove any dirt particles on the fruits’ surface. The fruits were chosen based on their colour, shape, and size for further analysis. The mature fruits could be obtained within two months after the flowering stage. The fully matured *P. macrocarpa* fruits were mostly chosen based on the observation of their red colour. The seed was removed, and the flesh was manually cut with a stainless-steel knife. The length and thickness of the fruits were maintained at 1.0 cm and 2.0 mm, respectively.

### 2.3. Sample Preparation

The fresh *P. macrocarpa* fruits were weighed (20 g) and placed on a flat tray. A total of 5 drying temperatures of 40, 50, 60, 70, and 80 °C were set in the oven to dry the samples at a constant velocity. The drying oven was preheated to the required temperatures prior to the loading process of the samples. The samples (20 g) were placed in a single layer across the tray of the dryer using a flat tray with the dimensions of 22 cm × 16 cm. Then, the sample tray was placed at the centre of the drying chamber to ensure a consistent drying process. The samples were removed and weighed at intervals during the drying process. The steps of removing, weighing, and replacing the *P. macrocarpa* fruits took about 1 min. The weight loss of the fruit samples was recorded using an analytical balance at 10 min interval for the first hour, followed by 20 min interval for the second hour, and 30 min interval for the next hour until a constant weight was achieved in 3 consecutive measurements. The oven-dried *P. macrocarpa* fruits were placed in a plastic container and stored at room temperature prior to further analysis.

### 2.4. Drying Kinetics

#### 2.4.1. Determination of Thin-Layer Mathematical Modelling

The moisture content of the samples was calculated using Equation (1):

(1) $$M=\frac{m_{t}-m_{dm}}{m_{dm}}$$

where M is the moisture content (g water/g dry matter), $m_{t}$ is the mass of the samples (g) at a specific time, and $m_{dm}$ is the mass of the dry weight.

The drying rate (DR) was determined by the following Equation (2):

(2) $$DR=\frac{M_{t}-M_{t+dt}}{d_{t}}$$

where $M_{t}$ is the moisture content at t time (g water/g dry matter), $M_{t+dt}$ is the moisture content at t+dt time (g water/g dry matter), and $d_{t}$ is the drying time (min).

Then, the experimental moisture content data were analysed to obtain a drying rate curve. The curve consists of the moisture ratio as the manipulated variable and time as the responding variable. The moisture content at different oven-drying temperatures was converted into a dimensionless moisture ratio using Equation (3):

(3) $$MR=\frac{M_{t}-M_{e}}{M_{i}-M_{e}}$$

where MR is the moisture ratio, $M_{t}$ is the moisture content at t time (g water/g dry matter), $M_{e}$ is the equilibrium moisture content, and $M_{i}$ is the initial moisture content.

The equilibrium moisture content was reached when the three consecutive weights of the product remained constant, signifying the completion of the drying process. Six kinetics models were utilised in this study to choose the most suitable model to represent the drying behaviour of *P. macrocarpa* fruits by fitting the experimental data to the prediction data. The six thin-layer models were extensively used to assess the drying kinetics of numerous food products. Table 1 presents the thin-layer models and their constant parameters.

The goodness of fit for the selected models was analysed based on statistical tools such as the correlation coefficient ($R^{2}$), root mean square error (RMSE), and chi-square ($\chi^{2}$). The best model should have the highest $R^{2}$ value, and the lowest RMSE and $\chi^{2}$ values [2]. The equations of the parameters are presented in Equation (4), Equation (5), and Equation (6), respectively:

(4) $$R^{2}=1-\left[\frac{\sum_{i=1}^{N}{\left(MR_{exp,i}-MR_{pre,i}\right)}^{2}}{\sum_{i=1}^{N}{\left(MR_{exp,i}-\bar{MR_{exp,i}}\right)}^{2}}\right]$$

(5) $$RMSE={\left[\sum_{i=1}^{N}\frac{1}{N}{\left(MR_{exp,i}-MR_{pre,i}\right)}^{2}\right]}^{\frac{1}{2}}$$

(6) $$\chi^{2}=\sum_{i=1}^{N}{\left(\frac{MR_{exp,i}-MR_{pre,i}}{MR_{pre,i}}\right)}^{2}$$

where N is the number of observations, $MR_{exp,i}$ and $MR_{pre,i}$ are the experimental and predicted dimensionless moisture ratios, respectively, and $\bar{MR_{exp,i}}$ is the mean value of the experimental dimensionless moisture ratio.

#### 2.4.2. Determination of Effective Moisture Diffusivity

The determination of the effective moisture diffusion coefficient during the drying process of *P. macrocarpa* fruits was measured using Fick’s second law [18]. The equation is expressed by Equation (7), whereas for a longer drying process, MR < 0.6, the equation is simplified by Equation (8):

(7) $$MR=\frac{8}{\pi^{2}}\sum_{n=1}^{\infty }\frac{1}{{\left(2n+1\right)}^{2}}exp\left(-{\left(2n+1\right)}^{2}\pi^{2}\frac{D_{eff}}{4L^{2}}t\right)$$

(8) $$ln\left(MR\right)=ln\left(\frac{8}{\pi^{2}}\right)-\left(\pi^{2}\frac{D_{eff}}{4L^{2}}t\right)$$

where $D_{eff}$, L, t, and n are the effective moisture diffusion coefficient $\left(m^{2}/s\right)$, half-thickness of the initial sample (m), drying time (s), and an integer value, respectively.

The $D_{eff}$ could be described by empirical data using the graph of ln(MR) versus the drying time (s), and the slope of the straight line from the plot as $-\left(\pi^{2}\frac{D_{eff}}{4L^{2}}t\right)$.

#### 2.4.3. Determination of Activation Energy

The activation energy was estimated by the Arrhenius equation [18] and accordingly expressed in Equation (9):

(9) $$D_{eff}=D_{0}exp\left(-\frac{E_{a}}{RT}\right)$$

where $D_{0}$ is the constant in the Arrhenius equation $\left(m^{2}/s\right)$, $E_{a}$ is the activation energy $\left(\frac{kJ}{\left(mol\;K\right)}\right)$, R is the universal gas constant $\left(\frac{kJ}{\left(mol\;K\right)}\right)$, and T is the temperature in degrees Kelvin (K).

The value of $E_{a}$ (kJ/mol) can be determined using exponential regression by plotting $D_{eff}$ versus 1/RT.

### 2.5. Estimation of Extraction Yield

The oven-dried *P. macrocarpa* fruits were ground into powder using an electric blender. An amount of 10 g of the grounded samples was weighed and put in the conical flask to mix with 200 mL ethanol (70%) at the ratio 1:20 g/mL of solid to solvent in the extraction process. The processes were initiated using ultrasound-assisted extraction, by setting the ultrasonic water bath at a frequency of 40 kHz and 320 W power output. Circulating hot water was used to maintain the temperature at 40 °C for 60 min. The extracted solutions were filtered using filter paper to remove the solid residues. Then, the filtered solutions were concentrated to dryness using a rotary evaporator. The filtered solutions were poured into a round-bottomed flask for the evaporation process at low pressure to remove the excess ethanol using the rotating vacuum evaporator. The temperature was set to 50 °C and after the process, the concentrated solutions were dried in an oven at 40 °C for 24 h to obtain the extraction yield. The extraction yield in percentage was obtained from Equation (10):

(10) $$\mathrm{Extraction}\;\mathrm{yield}\;\left(\%\right)=\frac{\mathrm{Dried}\;\mathrm{mass}\;\mathrm{of}\;\mathrm{crude}\;\mathrm{extract}\;\left(\mathrm{mg}\right)}{\mathrm{Mass}\;\mathrm{of}\;\mathrm{raw}\;\mathrm{material}\;\left(g\right)}\times 100$$

### 2.6. Estimation of Phenolics and Flavonoids

#### 2.6.1. Total Phenolic Content

The total phenolic content (TPC) of the oven-dried *P. macrocarpa* fruits using the F–C method was determined according to Ainsworth and Gillespie [19] with slight modifications. The reaction mixture was prepared by mixing 100 µL of crude extract (1 mg/mL concentration) with 500 µL of F–C reagent and 1.5 mL of 20% Na_2_CO_3_. The volume of the mixture was made up to 10 mL by immediately adding the pure water. Thus, the obtained coloured mixture was shaken well and incubated for 2 h in darkness. Then, the absorbance was measured at 765 nm against the blank using the UV–Vis spectrophotometer. A standard solution of gallic acid was used to obtain a calibration curve (y=0.0069x+0.0673) for the determination of TPC, which was expressed as the mg of gallic acid equivalent to 1 g of the dried sample (mg GAE/g).

#### 2.6.2. Total Flavonoid Content

The total flavonoid content (TFC) of the oven-dried *P. macrocarpa* fruits was determined according to Awang et al. [20] with minor modifications using an aluminium colorimetric assay. Firstly, 1 mL of crude extract (1 mg/mL concentration) was mixed with 1 mL of 2% AlCl_3_, and the reaction mixture was incubated in the dark for 15 min at room temperature. Then, the absorbance was measured spectrophotometrically at 430 nm against the blank. The TFC was expressed as the mg of rutin equivalent to 1 g of the dried sample (mg RE/g) using rutin as the standard solution based on the calibration curve (y=0.0589x−0.0277).

### 2.7. Estimation of Antioxidant Activity

The DPPH radical scavenging effect was estimated using the method described by Nithianantham et al. [21] with some alterations. Briefly, 1 mL of the crude extract (1 mg/mL concentration) was mixed with 1 mL of DPPH–methanolic solution (0.1 mM). The reaction mixture was incubated for 30 min in the dark at room temperature. The absorbance of the solution was measured at 517 nm using the UV–Vis spectrophotometer. Ascorbic acid served as a positive control for the effect comparison with treatments. The activity of the extract to scavenge the DPPH radical scavenging was formulated from the Equation (11):

(11) $$\mathrm{DPPH}\;\mathrm{radical}\;\mathrm{scavenging}\;\mathrm{activity}\;\left(\%\right)=\frac{A_{c}-A_{s}}{A_{c}}\times 100$$

where $A_{c}$ and $A_{s}$ are the control and sample absorbance, respectively.

### 2.8. Statistical Analysis

All experiments were conducted in triplicates. The statistical value of $R^{2}$, RMSE, and $\chi^{2}$ of the parameter models was estimated by Excel Solver (Microsoft Office 2019) for the thin-layer modelling analysis. Correlation analysis was performed via the Pearson correlation coefficient (R). The other experimental data were expressed as mean ± standard deviation. The data were analysed using SPSS (Version 28) as the statistics software. The results were further analysed by one-way analysis of variance (ANOVA), followed by a Tukey’s HSD test, with the level of statistical significance set at *p* < 0.05.

## 3. Results and Discussion

### 3.1. Effect of Drying Temperatures on Drying Times and Drying Rates

**Figure 3:**
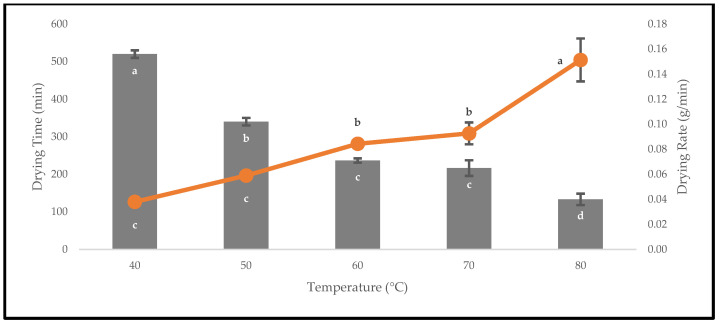
The drying times and drying rates of the oven-dried *P. macrocarpa* fruits at different temperatures. The values represent the means ± standard deviations of three replicates. Different letters (within a bar and line) indicate significant differences (one-way ANOVA, Tukey’s HSD test, *p* < 0.05).

The fresh fruits of *P. macrocarpa* were oven-dried to avoid chemical degradation and microbial contamination during storage. The drying rates of the oven-dried *P. macrocarpa* fruits were inversely proportional to the drying times up to 80 °C (Figure 3). The time taken for drying was 520 ± 10.00 min at 40 °C, whereas the time was shortened to 133.33 ± 15.28 min at 80 °C. In this study, the drying rates required to reach the equilibrium moisture content were 0.04 ± 0.00, 0.06 ± 0.00, 0.08 ± 0.00, 0.09 ± 0.01, and 0.15 ± 0.02 g/min at 40, 50, 60, 70, and 80 °C, respectively. The figure also showed that the drying rate increased with the higher drying temperatures. A higher drying temperature of 80 °C was found to reduce the drying time until a mass equilibrium of approximately 3.01 g was reached. This explanation was predicted due to the high heat transfer rate with the increased drying temperature, which resulted in an enhanced drying rate and a shortened time [22].

### 3.2. Effect of Drying Temperatures on Moisture Removal Rates

**Figure 4:**
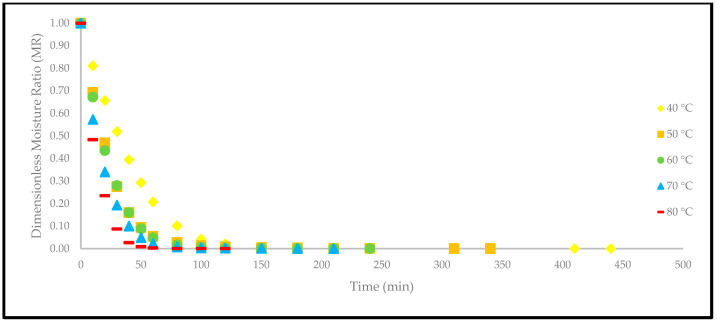
The drying curves of the oven-dried *P. macrocarpa* fruits at different temperatures.

Based on Figure 4, the drying curves of the oven-dried *P. macrocarpa* fruits at different temperatures showed a similar pattern. However, the different rate of those drying curves was seen (Figure 3). Based on the result, a higher temperature would increase the drying rate and shorten the time to reach the moisture equilibrium. Further increment of the temperature up to 80 °C did significantly shorten the drying duration. Hence, the temperature of 40 °C took the longest drying time to achieve an equilibrium of the moisture rate, whereas the temperature of 80 °C obtained the shortest drying time of all other temperatures. This phenomenon demonstrates the parabolic curve that could be observed for the correlation between the drying duration and temperature. Furthermore, the MR tends to go lower as the allocated time to dry the food increases. A relatively rapid trend in the moisture removal rate was seen at the beginning of the drying process. Nevertheless, it gradually decreased as the drying process continued. Moreover, most of the predicted data were clustered around the straight line, demonstrating that the experimental model was suitable to explain the single-layer drying curve of the *P. macrocarpa* fruits.

A higher drying temperature was associated with the increase in the drying rate in most situations and, consequently, induced more heat transfer to the sample. This state will increase the diffusion of moisture to the interior and exterior of materials. It was also previously reported that the moisture removal rate was accelerated at higher oven-drying temperatures [23]. Additionally, the lack of a constant drying rate may be explained by the thin-layer arrangement and the increased flow of the drying agent, which rapidly accelerated the evaporation process and circumvented the saturation state of the material. Recent studies have also revealed that the constant-rate period was absent from the drying processes of several fruits, such as in amla [24], pumpkin [25], red dragon fruit [26], strawberry [27], and pomegranate [28]. This condition is due to the extremely short period of the drying process [29].

### 3.3. Effect of Drying Temperatures on Thin-Layer Drying Models

Six thin-layer drying models, namely Lewis, Page, Henderson and Pabis, Logarithmic, two-term exponential, and Midilli and Kucuk were applied to describe the drying process of *P. macrocarpa* fruits in this study. The model with the highest $R^{2}$ value, and the lowest RMSE and $\chi^{2}$ values was selected as the criteria for the goodness of fit. Hence, Table 2 shows the result of fitting the experimental data to the thin-layer drying models.

From Table 2, it was found that the $R^{2}$ values varied as follows: 0.994 to 0.999 for the Lewis model, 0.999 to 1.000 for the Page model, 0.995 to 0.999 for the Henderson and Pabis model, 0.994 to 0.999 for the two-term exponential model, 0.996 to 1.000 for the Logarithmic model, and 0.999 to 1.000 for the Midilli and Kucuk model. When the values of $R^{2}$ for the models are more significant than the acceptable threshold of 0.90, it indicates a good fit [30]. Resultantly, it was shown that the value of $R^{2}$ is close to 1 for all drying temperatures, indicating that they are well correlated. In addition, the RMSE values at the 5 temperatures were ranged as follows: 0.240 × $10^{-4}$ to 2.797 × $10^{-4}$ for the Lewis model, 0.145 × $10^{-4}$ to 0.324 × $10^{-4}$ for the Page model, 0.237 × $10^{-4}$ to 2.284 × $10^{-4}$ for the Henderson and Pabis model, 0.240 × $10^{-4}$ to 3.092 × $10^{-4}$ for the two-term exponential model, 0.209 × $10^{-4}$ to 2.057 × $10^{-4}$ for the Logarithmic model, and 0.143 × $10^{-4}$ to 0.297 × $10^{-4}$ for the Midilli and Kucuk. Another critical parameter to determine the validity of the models is the chi-square ($\chi^{2}$). The lowest $\chi^{2}$ ranges were recorded as follows: 0.248 × $10^{-4}$ to 3.910 × $10^{-4}$ for the Lewis model, 0.103 × $10^{-4}$ to 0.721 × $10^{-4}$ for the Page model, 0.818 × $10^{-4}$ to 4.959 × $10^{-4}$ for the Henderson and Pabis model, 0.671 × $10^{-4}$ to 38.081 × $10^{-4}$ for the two-term exponential model, 0.000 × $10^{-4}$ to 0.088 × $10^{-4}$ for the Logarithmic model, and 0.028 × $10^{-4}$ to 0.138 × $10^{-4}$ for the Midilli and Kucuk. Furthermore, it was discovered that the constant values of each model at the same drying temperature were fairly similar.

Overall, the value of $R^{2}$ obtained at 60 °C and 70 °C for the Midilli and Kucuk model was at 1.000, whereas the rest were at 0.999. Furthermore, the RMSE and $\chi^{2}$ values obtained for the Midilli and Kucuk model were also lower than the rest of the models. Considering the values of $R^{2}$, RMSE, and $\chi^{2}$, the Midilli and Kucuk model fitted well in the drying curve to predict the drying behaviour of the oven-dried *P. macrocarpa* fruits. Similar findings previously concluded that the Midilli and Kucuk model was the most well-fitted model to describe the drying behaviour of fruits, such as strawberry [31], papaya [32], and goldenberry [33]. Both the drying constant (k) and the empirical constant (a) for the oven-dried *P. macrocarpa* fruits described by Midilli and Kucuk were shown to be significantly increased with the increasing temperature. Therefore, as the drying air temperature rises, so does the model’s constant.

**Figure 5:**
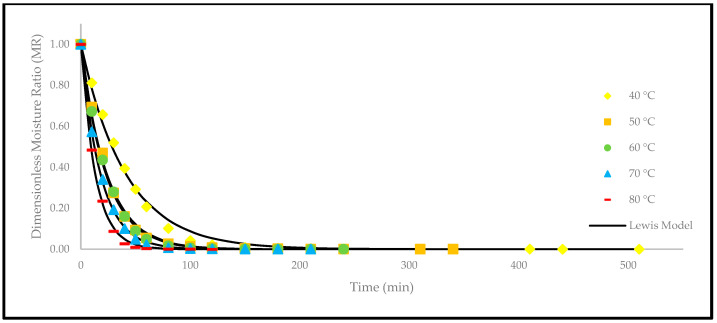
The drying curves of the oven-dried *P. macrocarpa* fruits with experimental and predicted data based on the Lewis model at different temperatures.

**Figure 6:**
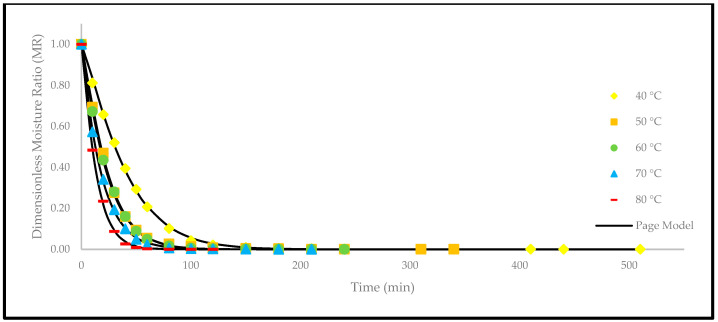
The drying curves of the oven-dried *P. macrocarpa* fruits with experimental and predicted data based on the Page model at different temperatures.

**Figure 7:**
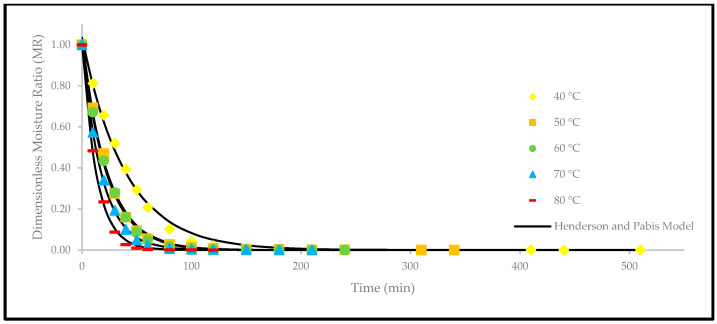
The drying curves of the oven-dried *P. macrocarpa* fruits with experimental and predicted data based on the Henderson and Pabis model at different temperatures.

**Figure 8:**
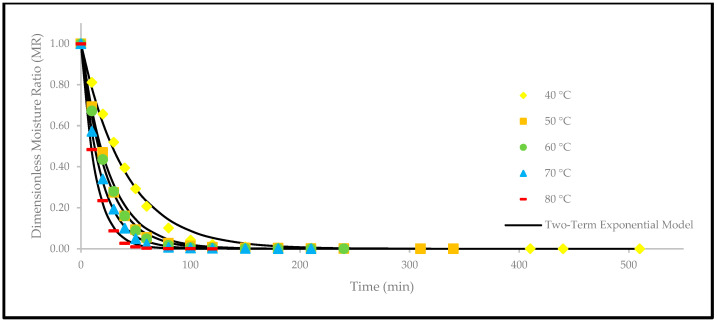
The drying curves of the oven-dried *P. macrocarpa* fruits with experimental and predicted data based on the two-term exponential model at different temperatures.

**Figure 9:**
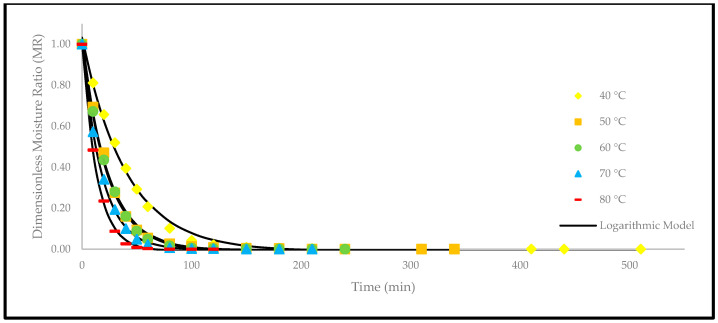
The drying curves of the oven-dried *P. macrocarpa* fruits with experimental and predicted data based on the Logarithmic model at different temperatures.

**Figure 10:**
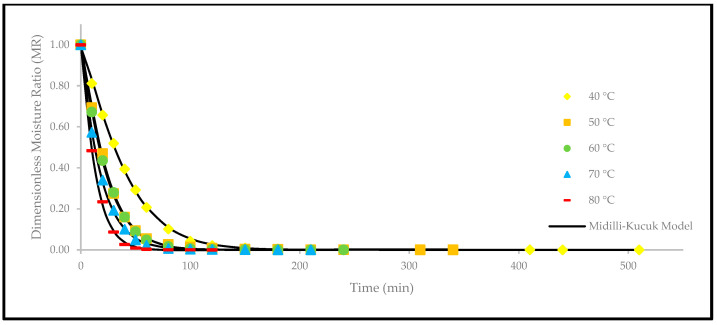
The drying curves of the oven-dried *P. macrocarpa* fruits with experimental and predicted data based on the Midilli and Kucuk model at different temperatures.

Apart from that, a comparison of the experimental and predicted MR values with the drying time are shown in Figure 5, Figure 6, Figure 7, Figure 8, Figure 9 and Figure 10. The MR values were predicted by the Lewis, Page, Henderson and Pabis, two-term exponential, Logarithmic, and Midilli and Kucuk models at different temperatures, respectively.

In summary, the Midilli and Kucuk model is one of the more promising models to characterise the drying kinetics of various fruits and vegetables. As a result, the Midilli and Kucuk model is the most suitable drying model in this study that describes the drying kinetics of *P. macrocarpa* fruits during oven-drying at temperatures of 40, 50, 60, 70, and 80 °C. The selection was supported by the statistical results and graphical curve models obtained throughout the study. As a result, it can be inferred that modelling the thin-layer drying kinetics of fruits and vegetables will provide the necessary information concerning the air velocity, ideal drying time, relative humidity, storage conditions, and temperature [29]. With all these data, a dryer with greater efficiency can be designed, and its process could be potentially optimised, thereby reducing postharvest losses.

### 3.4. Effect of Drying Temperatures on Effective Moisture Diffusivity and Activation Energy

**Figure 11:**
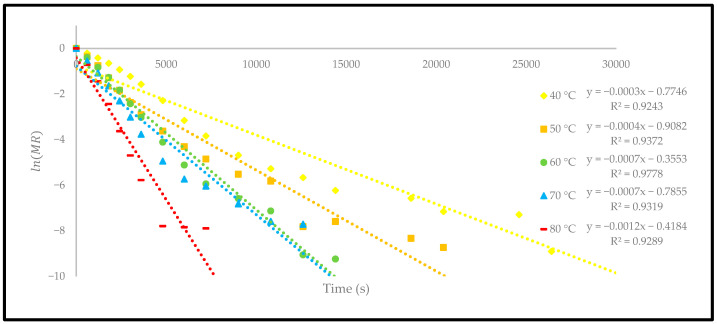
The relationship between ln(MR) and time of the oven-dried *P. macrocarpa* fruits.

The effective moisture diffusivity ($D_{eff}$) was calculated from the plots of ln(MR) against the drying time (s) at different temperatures (Figure 11). The slope of each linear regression plot was applied to estimate the $D_{eff}$ coefficient. The change in $D_{eff}$ coefficient at different temperatures is presented in Table 3.

The $D_{eff}$ coefficient showed a significant increase in temperature from 1.22 × $10^{-8}$ to 4.86 × $10^{-8}$ $m^{2}/s$. The finding of this study is consistent with the previously reported studies, which recorded within the general range of $10^{-6}$ to $10^{-12}$ $m^{2}/s$ for typical food-drying processes [34]. Different drying methods were studied to investigate the change in the effective moisture diffusivity process to dry the different products. However, different drying methods, either using a thermal convection oven or a hot-air dryer, also indicated an increase in the $D_{eff}$ coefficient when the temperature was increased [23]. This explanation is most likely because a high temperature increases the heat absorption in material and consequently increases the mass transfer [35]. Therefore, the $D_{eff}$ coefficient is highly dependent on the increase in the drying temperature. At 80 °C, the moisture content of the oven-dried *P. macrocarpa* fruits peaked since a higher temperature would quicken the evaporation of water molecules on the surface of the *P. macrocarpa* material.

**Figure 12:**
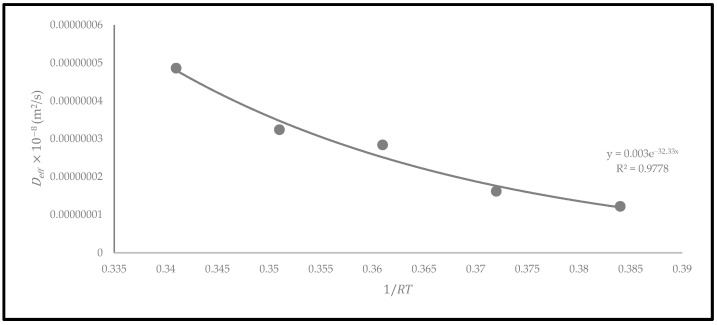
The relationship between $D_{eff}$ and 1/RT based on the Arrhenius model of oven-dried *P. macrocarpa* fruits.

The activation energy ($E_{a}$) represents the minimum energy required to start the removal of moisture from a material. The Arrhenius equation was used to plot the exponential regression graph of $D_{eff}$ against 1/RT [29]. This equation is essential for determining the activation energy of the oven-dried *P. macrocarpa* fruits in accordance with the drying temperature [29]. Based on the exponential regression plot (Figure 12), the activation energy of the oven-dried *P. macrocarpa* was determined at 32.33 kJ/mol using a convection drying oven. This condition indicated that more energy is needed to separate moisture from the material during the drying process. According to the scientific literature, more than 90% of the activation energy required for drying a food product ranged between 14.42 and 43.26 kJ/mol [29]. The activation energy obtained in this study is also comparable to the previous studies on some food products such as banana slices (32.65 kJ/mol) [36] and pumpkin (33.15 kJ/mol) [37]. Nevertheless, the difference may be attributable to the drying techniques, materials, and operating settings during the drying process.

### 3.5. Effect of Drying Temperatures on Extraction Yield

**Figure 13:**
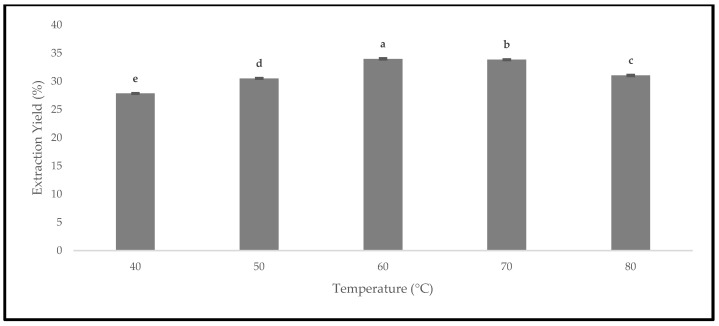
The extraction yield of the oven-dried *P. macrocarpa* fruits at different temperatures. The values represent the means ± standard deviations of three replicates. Different letters (within a bar) indicate significant differences (one-way ANOVA, Tukey’s HSD test, *p* < 0.05).

The quality of the oven-dried *P. macrocarpa* fruits was evaluated based on the extraction yield. The dried fruits could lead to a higher extraction yield due to the reduced water activity [38]. Therefore, Figure 13 presents the extraction yield of the oven-dried *P. macrocarpa* fruits at different temperatures. All the drying temperatures demonstrated a significant difference in the extraction yield at *p* < 0.05. The extraction yield from the oven-dried *P. macrocarpa* fruits at 40 °C is 27.86 ± 0.02%. At 50 °C, the extraction yield was slightly increased with the value of 30.54 ± 0.00%, followed by 60 °C with the value of 33.99 ± 0.05%. Similarly, Che Sulaiman et al. [39] found that the drying temperature at 60 °C exhibited the highest extraction yields for *Clinacanthus nutans* leaves compared to other temperatures. Nonetheless, the extraction yield steadily decreased at 70 and 80 °C, with the value of 33.86 ± 0.01% and 31.06 ± 0.03%, respectively. This result was in accordance with the findings of Che Sulaiman et al. [39] that stated a more extended extraction period at 80 °C could reduce the extraction yield because high temperatures could promote oxidation and degradation of the target chemicals. Plus, the heat-sensitive nature of the bioactive chemicals resulted in a considerable reduction in the yield when the temperature was raised to 80 °C [39].

Generally, the increase in the temperature can decrease the viscosity and significantly increase the diffusion rate [40]. Hence, a higher drying temperature could possibly increase the extraction yield. The oven-dried *P. macrocarpa* fruits at 60 °C produced the highest extraction yield in this study, while the temperature of 40 °C produced the lowest extraction yield. Simultaneously, the increase in the extraction yield resulted in a higher phytochemical content, such as the phenolic compound. Another study conducted by Fikselová [41] stated that the extracted efficiency of carotenes from carrots was achieved at an oven temperature of 60 °C. In contrast, maintaining a minimal temperature, such as 60 °C, produced the highest yields. Hence, it could be summarised from these results that the drying temperature during the extraction process would influence the extraction yield.

### 3.6. Effect of Drying Temperatures on Phenolics and Flavonoids

**Figure 14:**
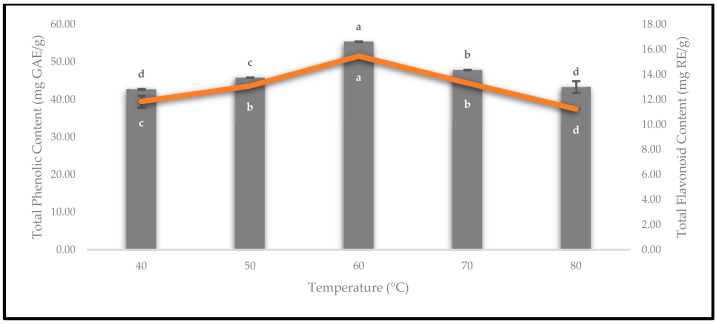
The TPC and TFC of the oven-dried *P. macrocarpa* fruits at different temperatures. The values represent the means ± standard deviations of three replicates. Different letters (within a bar and line) indicate the significant differences (one-way ANOVA, Tukey’s HSD test, *p* < 0.05).

The quality of the oven-dried *P. macrocarpa* fruits was evaluated by quantifying the values of the TPC and TFC. The TPC and TFC were estimated using gallic acid and rutin standard calibration curves, respectively. Thus, the results of the TPC (bar) and TFC (line) are presented in Figure 14. Based on the TPC results, drying at 40 °C (42.75 ± 0.09 mg GAE/g) yielded the lowest concentration of phenolics and was followed by 50 °C (45.87 ± 0.03 mg GAE/g). According to Cheng et al. [42], low-temperature heat treatments did not destroy the polyphenol oxidase enzymes, such as drying at temperatures below 55 °C. Conversely, the oven-dried *P. macrocarpa* fruits drying at 60 °C recorded the highest TPC, which was 55.39 ± 0.03 mg GAE/g. Similarly, a previous study by Muthukumar et al. [43] revealed that the highest yield of TPC was retained after the drying process of black ginger at 60 °C. Meanwhile, the oven-dried *P. macrocarpa* fruits at 70 and 80 °C inversely decreased the TPC from 47.90 ± 0.04 to 43.36 ± 1.57 mg GAE/g, respectively. A previous study by Izli et al. [44] recorded that the TPC on kumquat fruits decreased when the drying temperature changed from 70 to 80 °C. This decrease in the TPC could be due to the high temperature during the drying treatment that led to the decomposition of the heat liable phenolic compounds [44].

Based on Figure 14, the result of the TFC for the oven-dried *P. macrocarpa* fruits at 60 °C (15.47 ± 0.00 mg RE/g) was the highest value compared to the others. Kessy et al. [45] found that the highest TFC was at 60 °C for oven-dried litchi pericarps. Meanwhile, the lowest TFC in the oven-dried *P. macrocarpa* fruits was observed at an 80 °C drying temperature (11.24 ± 0.01 mg RE/g). According to Kessy et al. [45], the litchi pericarps that were subjected to a hot-air oven at temperatures higher than 60 °C considerably lost the TFC due to thermal deterioration. Sharma et al. [46] also suggested that this condition might be due to the degradation of flavonoids, as a result of the increased temperature. It also depends on the structure of the flavonoids [46]. Additionally, drying at 40, 50, and 70 °C for the oven-dried *P. macrocarpa* fruits exhibited 11.83 ± 0.47, 13.08 ± 0.08, and 13.32 ± 0.05 mg RE/g, respectively.

### 3.7. Effect of Drying Temperatures on Antioxidant Activity

**Figure 15:**
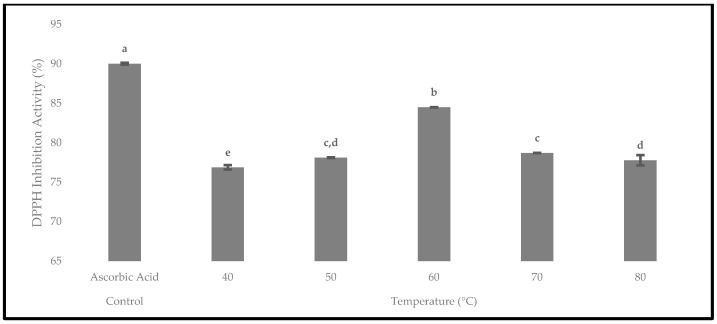
The antioxidant activity of the oven-dried *P. macrocarpa* fruits using the DPPH inhibition activity at different temperatures. The values represent the means ± standard deviations of three replicates. Different letters (within a bar) indicate significant differences (one-way ANOVA, Tukey’s HSD test, *p* < 0.05). Ascorbic acid was expressed as a positive control.

The antioxidant activity of the oven-dried *P. macrocarpa* fruits was evaluated using the DPPH assay. The presence of antioxidants would reduce the DPPH radical into the DPPH-H molecule, causing the decolourisation of the violet solution into the yellow colour and a decrease in the absorbance at 517 nm [47]. Hence, the DPPH inhibition activity at different temperatures is displayed in Figure 15. The results showed that the DPPH inhibition activity was higher when the drying temperatures were consistently increased from 40 to 50 °C, with the total values of 76.88 ± 0.28% and 78.11 ± 0.04%, respectively. Then, it was found that the oven-dried *P. macrocarpa* fruits at 60 °C contained the highest DPPH inhibition (antioxidant activity) with the value of 84.49 ± 0.02%. The result of the current study was in line with Muthukumar et al. [43], who reported that the drying of black ginger in an electrical dryer at 60 °C resulted in a maximum radical scavenging activity. Nonetheless, the DPPH inhibition activity gradually decreased from 70 °C (78.69 ± 0.03%) to 80 °C (77.77 ± 0.65%). Similarly, Krishnan et al. [48] found that the antioxidant activity on elephant apple slices decreased at 80 °C. In short, the oven-dried *P. macrocarpa* fruits at 40 °C had the lowest antioxidant activity.

Provenly, 60 °C was the most significant drying temperature at *p* < 0.05 for the oven-dried *P. macrocarpa* fruits in the TPC, TFC, and DPPH inhibition activity. Similarly, Kaur et al. [49] revealed that convective hot-air drying at 60 °C was the most effective temperature to maintain the most bioactive components in sweet peppers and tomatoes. Consequently, based on the findings, the optimal temperature for the oven-dried *P. macrocarpa* fruits was claimed to be 60 °C to achieve the maximal TPC, TFC, and antioxidant activity. Aryal et al. [50] mentioned that the antioxidant activity of plant materials is closely related to the presence of phenolic and flavonoid compounds. Thus, a plant extract’s polyphenol level is frequently linked to its antioxidant properties. Hence, the correlation between the antioxidant activity, phenolics, and flavonoids of the oven-dried *P. macrocarpa* fruits was evaluated (Table 4).

Briefly, all the correlation coefficient values of the oven-dried *P. macrocarpa* fruits demonstrated a strong correlation between DPPH-TPC (R = 0.97) and DPPH-TFC (R = 0.90). According to Aryal et al. [50], higher TPC and TFC values would also cause higher antioxidant activity. Hence, the findings proved that the antioxidant activity is strongly correlated with the presence of phenolics and flavonoids.

## 4. Conclusions

In accordance with our research, a mathematical model that best captures the behaviour of *P. macrocarpa* fruits dried in an oven has been identified. The findings revealed that a higher drying temperature is linked with a shortened drying time and a quicker rate of moisture removal. In addition, the drying process consisted entirely of the initial and falling-rate periods without a constant-rate period. The Midilli and Kucuk model was the most suitable model that could describe the thin-layer drying process using the oven-drying method. It was also shown that this model has the highest $R^{2}$ (>0.999), and the lowest RMSE (<0.297 × $10^{-4}$) and $X^{2}$ (<0.138 × $10^{-4}$). The effective moisture diffusivity (ranges from 1.22 × $10^{-8}$ to 4.86 × $10^{-8}$ ${(m}^{2}/s)$ and activation energy (32.33 kJ/mol) of the material were also computed.

Moreover, the extraction yield from the oven-dried *P. macrocarpa* fruits at 60 °C (33.99 ± 0.05%) was the highest value among others. The maximum TPC and TFC exhibited in the oven-dried *P. macrocarpa* fruits were at 60 °C with the total values of 55.39 ± 0.03 mg GAE/g and 15.47 ± 0.00 mg RE/g, respectively. In addition, the antioxidant activity of *P. macrocarpa* fruits possessed a strong inhibition activity after the drying process at 60 °C (84.49 ± 0.02%). Based on the correlation study, the antioxidant activity was found to be strongly linked with the content of the phenolics and flavonoids in the oven-dried *P. macrocarpa* fruits. These findings summarised that the oven-dried *P. macrocarpa* fruits at 60 °C showed an effective retention of bioactive components. Further investigation on oven-dried *P. macrocarpa* fruits is crucial to prolong the shelf life and retain bioactive compounds as functional ingredients for foods and nutraceuticals with high therapeutic values. Based on the limitation of the study, a specific range of temperatures, i.e., 40–80 °C, was only emphasised. Evaluation of novel extraction techniques for boosting the phenolics and flavonoids in *P. macrocarpa* fruits with enhanced management and storage at an industrial level for potential commercialisation is recommended for further study.

## Figures and Tables

**Table 1:** Thin-layer mathematical modelling of oven-dried *P. macrocarpa* fruits.

Model	Model Expression	References
Lewis	MR=exp(−kt)	[10]
Page	$MR=exp\left(-kt^{n}\right)$	[11]
Henderson and Pabis	MR=aexp(−kt)	[12]
Two-term exponential	MR=aexp(−kt)+(1−a)exp(−kat)	[13,14]
Logarithmic	MR=aexp(−kt)+b	[15,16]
Midilli and Kucuk	$MR=aexp\left(-kt^{n}\right)+bt$	[14,17]

*k* is the drying rate constant (min^−1^), *a*, *b*, *k*, and *n* are the constant, and *t* is the drying time (min).

**Table 2:** The statistical analysis of different thin-layer drying models of the oven-dried *P. macrocarpa* fruits at different temperatures.

Model	Temperature (°C)	Constant	$R^{2}$	RMSE $\;(10^{-4})$	$\chi^{2}$ $\;(10^{-4})$
Lewis	40	k = 0.024	0.994	2.797	1.258
	50	k = 0.042	0.996	1.697	0.248
	60	k = 0.044	0.998	1.041	1.503
	70	k = 0.056	0.999	0.240	0.671
	80	k = 0.075	0.999	0.782	3.910
Page	40	k = 0.011	1.000	0.324	0.228
		n = 1.202			
	50	k = 0.024	0.999	0.234	0.637
		n = 1.173			
	60	k = 0.029	1.000	0.145	0.103
		n = 1.122			
	70	k = 0.05	1.000	0.174	0.210
		n = 1.035			
	80	k = 0.056	0.999	0.307	0.721
		n = 1.102			
Henderson and Pabis	40	a = 1.034	0.995	2.284	4.320
		k = 0.025			
	50	a = 1.023	0.997	1.486	1.569
		k = 0.043			
	60	a = 1.015	0.998	0.929	3.034
		k = 0.044			
	70	a = 1.002	0.999	0.237	0.818
		k = 0.056			
	80	a = 1.005	0.999	0.765	4.959
		k = 0.076			
Two-term exponential	40	a = 1.000	0.994	2.797	1.258
		k = 0.024			
	50	a = 1.000	0.993	3.092	38.081
		k = 0.039			
	60	a = 1.000	0.998	1.041	1.503
		k = 0.044			
	70	a = 1.000	0.999	0.240	0.671
		k = 0.056			
	80	a = 1.000	0.999	0.782	3.910
		k = 0.075			
Logarithmic	40	a = 1.041	0.996	2.057	0.000
		k = 0.025			
		b = −0.009			
	50	a = 1.026	0.997	1.437	0.088
		k = 0.043			
		b = −0.004			
	60	a = 1.021	0.998	0.795	0.000
		k = 0.043			
		b = −0.007			
	70	a = 1.005	1.000	0.209	0.000
		k = 0.055			
		b = −0.003			
	80	a = 1.013	0.999	0.560	0.000
		k = 0.074			
		b = −0.009			
Midilli and Kucuk	40	a = 0.989	0.999	0.290	0.030
		k = 0.010			
		n = 1.222			
		b = 0.000			
	50	a = 0.998	0.999	0.224	0.138
		k = 0.023			
		n = 1.178			
		b = 0.000			
	60	a = 0.998	1.000	0.143	0.028
		k = 0.029			
		n = 1.125			
		b = −0.000			
	70	a = 0.998	1.000	0.172	0.075
		k = 0.050			
		n = 1.036			
		b = −0.000			
	80	a = 0.999	0.999	0.297	0.120
		k = 0.056			
		n = 1.099			
		b = −0.000			

**Table 3:** Values of $D_{eff}$ of oven-dried *P. macrocarpa* fruits at different temperatures.

Temperature (°C)	$\mathrm{Slope}\;(k_{0})$	$D_{eff}\;\times \;10^{-8}{\;(m}^{2}/s)$	$R^{2}$
40	−0.0003	1.22	0.9243
50	−0.0004	1.62	0.9372
60	−0.0007	2.84	0.9778
70	−0.0008	3.24	0.9319
80	−0.0012	4.86	0.9289

**Table 4:** The Pearson correlation between the antioxidant activity, phenolics, and flavonoids of the oven-dried *P. macrocarpa* fruits.

Antioxidant Activity	TPC		TFC	
	*R*	*p*-Value	*R*	*p*-Value
DPPH	0.97 **	0.00	0.90 **	0.00

** Correlation is significant at the 0.01 level (2-tailed).

## Data Availability

The data used to support the findings of this study can be made available by the corresponding author upon request.
